# Selected Clinical Features Fail to Predict Inflammatory Gene Expressions for TNF-α, TNFR1, NSMAF, Casp3 and IL-8 in Tendons of Patients with Rotator Cuff Tendinopathy

**DOI:** 10.1007/s00005-021-00610-z

**Published:** 2021-03-08

**Authors:** Slawomir Struzik, Bozena Czarkowska-Paczek, Aleksandra Wyczalkowska-Tomasik, Paweł Maldyk, Leszek Paczek

**Affiliations:** 1 Department of Orthopedics and Traumatology, Medical University of Warsaw, Warsaw, Poland; 2 Department of Clinical Nursing, Medical University of Warsaw, E. Ciolka 27, 01-445 Warsaw, Poland; 3 Department of Immunology, Transplantology and Internal Diseases, Medical University of Warsaw, Warsaw, Poland

**Keywords:** Rotator cuff tendinopathy, Cytokines, TNF pathway, Interleukin 8, Caspase-3

## Abstract

The pathophysiology of rotator cuff tendinopathy is not fully understood, particularly in terms of the local inflammatory process. This study aimed to investigate the expression of selected molecules in the tumour necrosis factor (TNF)-α transduction pathway, including TNF-α, TNF receptor 1 (TNFR1), neutral sphingomyelinase activation associated factor (NSMAF), caspase 3 (Casp3), and interleukin (IL)-8, in patients with rotator cuff tendinopathy that had undergone surgical treatment. We included 44 participants that underwent arthroscopy, due to rotator cuff tendinopathy. Samples from the injured tendon were collected during arthroscopy, and RT-PCR was performed to determine gene expression. Pearson correlation analyses or *U*-Mann–Whitney test were performed to identify associations with the following parameters: sex, age at admission, body mass index, the presence of night pain, previous treatment (nonsteroidal anti-inflammatory drugs and/or steroids), medical history of the shoulder injury, upper subluxation of the humeral head, and the number of tendons injured. RT-PCR showed that the selected pro-inflammatory factors involved in the TNF-α signalling pathway expression levels were expressed in the tendon tissues. However, the levels of expression varied from patient to patient. Variations were over 250-fold for TNF-α, about 130-fold for TNFR1, NSMAF, and Casp3, and 1000-fold for IL-8. We could not confirm that any of the clinical parameters investigated were associated with the level of gene expression in the TNF-α pathway and IL-8.

## Introduction

Rotator cuff (RC) tendinopathy is one of the principal causes of shoulder pain. Tendinopathy is characterised by pain in and around the tendon associated with repetitive sports or daily living activities. The risk of RC tendinopathy increases with age, however, the aging process is fully natural and distinct from tendinopathy. RC tendinopathy affects people performing sport or repetitive activities resulting from everyday living. (Spargoli 2018; Zabrzynski et al. 2018).

The aetiology of RC tendinopathy that leads to a partial or full-thickness tear is complex, and the pathophysiology remains unclear (Pandey and Jaap Willems 2015). Cuff tears are either traumatic or degenerative, resulting from extrinsic or intrinsic factors (Spargoli 2018). Extrinsic factors consist of various anatomical and biomechanical alterations that lead to narrowing of the subacromial space and mechanical compression of RC tendons. Intrinsic factors consist of multiple potential mechanisms. One is degenerative-microtrauma, where age-related collagen fibre disorganisation and structural changes reduce the ability of the tendon to withstand repetitive microtraumas that could lead to a partial- or full-thickness tear. Other mechanisms include harmful oxidative stress around the tendon, induced by repetitive injury, followed by a reparative process or a change in vascularisation (Cipollaro et al. 2019; Pandey and Jaap Willems 2015). However, contradictory results have been presented. Some studies described areas with lower vascularisation in tendinopathy, and others described abundant neovascularisation in tendinopathy. Abundant neovascularisation could result in crowding, which could weaken collagen fibres and increase the susceptibility to injury (Spargoli 2018). Zabrzynski et al. (2020) revealed that the presence of neovessels is less important than the mediators and cytokines in injured tendons, what is in line with some evidence that inflammation was present in tendinopathy; however, this issue remains controversial. Inflammatory cells were found in RC tears, but the infiltration was inversely correlated with the size of the tears (Neal et al. 2010). Some studies reported an increase in inflammatory mediators associated with RC tendinopathy, but others found no correlation between increases in inflammatory cytokines and histological changes in tendinopathy. No study has excluded the possibility that degeneration and inflammation might occur simultaneously and act in concert in the pathophysiology of RC tendinopathy (Cipollaro et al. 2019; Longo et al. 2011; Zabrzynski et al. 2017). Thus, the role of inflammation in RC tendinopathy remains to be defined.

Tumour necrosis factor (TNF)-α is one of the most important pro-inflammatory cytokine and a key mediator of acute and chronic inflammation. It induces its own secretion and it also stimulates the production of other inflammatory cytokines and chemokines. TNF-α is also involved in the initiation of apoptosis and necrosis (Chu 2013). There are very limited data regarding the involvement of TNF-α pathway into pathophysiology of RC tendinopathy, therefore the present study aimed to investigate the gene expression for selected molecules in the (TNF-α) transduction pathway, including TNF-α, TNF receptor 1 (TNFR1), neutral sphingomyelinase activation associated factor (NSMAF), and caspase 3 (Casp3) and interleukin (IL)-8, in patients with RC tendinopathy that underwent surgical treatment.

## Materials and Methods

This study included 44 patients admitted to the Department of Orthopaedics and Traumatology, Child Jesus Hospital in Warsaw, Poland. The inclusion criterion was a diagnosis of RC tendinopathy with an indication for surgical treatment (arthroscopy). The exclusion criteria were recommendations for conservative treatment or disqualification from general anaesthesia. The diagnosis of RC tendinopathy was confirmed with the standard clinical examination, including an X-ray, an ultrasound examination, and/or magnetic resonance imaging, and an assessment of the range of motion in the shoulder. The shoulder range of motion was defined as the ability to move each shoulder in different directions without major joint pain. The surgeries (arthroscopies) were performed after administering general anaesthesia combined with conduction anaesthesia of the brachial plexus, between the inclined muscles. After the arthroscope was inserted and damage to the RC was confirmed, a 3 × 3 mm fragment of tendon was collected, and immediately placed at − 80 °C. All surgeries and sample collections were performed by the same surgeon.

For each participant, we collected data on sex, age at admission, body mass index (BMI), duration of complaints (in months), the presence of night pain, previous treatment (nonsteroidal anti-inflammatory drugs [NSAIDs] and/or steroids), medical history of the shoulder injury, upper subluxation of the humeral head, and the number of tendons injured.

### Analytical Methods

Tissue homogenisation was performed in a TissueLyser (Qiagen, Germany) with QIAzol Lysis Reagent (Qiagen, Germany) and steel balls (5 mm in diameter). Homogenisation was performed at room temperature at 25 Hz for 10 min. Total RNA was isolated from the samples with the EZ1 RNA Universal Tissue Kit (Qiagen, Germany) in a BioRobot EZ1 (Qiagen, Germany), according to the manufacturer’s instructions. After RNA was verified qualitatively and quantitively with a NanoDrop ND-1000 camera (NanoDrop Technologies, Wilmington, Delaware, USA), the nucleic acids were stored at − 80 °C. Gene expression was examined with real-time PCR (RT-PCR) carried out with the ABI 7500 thermocycler (Applied Biosystems, Waltham, MA, USA). To each well of a 96-well Optical Reaction Plate (Applied Biosystems), we added the reaction mixture, which included TaqMan® RNA-to-Ct™ 1-Step Kit (Thermo Fisher Scientific) and appropriately selected primers, in the TaqMan Gene Expression Assay (Thermo Fisher Scientific). Reactions included primers specific for the following genes: TNF-α, TNFR1, NSMAF, Casp3, IL-8, and glyceraldehyde 3-phosphate dehydrogenase (GAPDH, internal control gene). Next, total RNA was added to the reaction mixture in all wells, except a no template control (NTC), which received water (Sigma-Aldrich, Saint Louis, MI, USA) instead of RNA. The RT-PCR was performed according to the protocol recommended by Applied Biosystems (USA). The test samples and control were assayed in duplicate, and the NTC was assayed in triplicate. Gene expression levels were calculated with the comparative cycle threshold (Ct) method. Expression was evaluated as the relative expression level, where the Ct of each sample was normalised to the Ct of GAPDH as follows: deltaCt (dCt) = Ct research gene – Ct GAPDH.

### Statistical Analysis

All calculations were performed with STATSTICA 12.0. and R. The variables were analysed with basic descriptive statistics, including cardinality (*N*), arithmetic mean, median, minimum (min), maximum (max), lower (Q1) and upper (Q3) quartiles, and standard deviation (SD). The non-parametric Mann–Whitney *U* test or Fisher test were used to compare continuous variables. The Pearson test was used to analyse correlations between the investigated parameters. The assumed level of significance was *p* < 0.05.

## Results

Among the 44 participants, 24 were females (54.5%) and 20 were males (45.5%). The demographic and clinical characteristics of the study group are shown in Table 1.

**Table 1:** Demographic and clinical characteristics of patients with rotator cuff tendinopathy

Parameter	Unit/category	Patients *N* (%) unless otherwise indicated
Age (years)	*N*	44
	Mean (SD)	59.02 (10.36)
	Median (IQR)	58 (53–63)
	Range	37–85
Sex	Female	24 (54.5)
	Male	20 (45.5)
BMI (kg/m^2^)	*N*	44
	Mean (SD)	33.09 (32.78)
	Median (IQR)	27.51 (25.26–31.44)
	Range	20.2–243.6
Duration of complaints (months)	*N*	44
	Mean (SD)	23.5 (26.28)
	Median (IQR)	12 (6–24)
	Range	2–120
NSAID use	No	4 (9.1)
	Periodically	33 (75)
	Permanently	7 (15.9)
Steroid use	Yes	13 (29.5)
	No	31 (70.5)
Previous rehabilitation	Yes	40 (93)
	No	3 (7)
Medical history of the injury	Yes	18 (40.9)
	No	26 (59.1)
Night pain	No	3 (6.8)
	Periodically	25 (56.8)
	Permanently	16 (36.4)
Upper subluxation of humeral head	Yes	9 (20.5)
	No	35 (79.5)
Number of damaged tendons	1	19 (43.2)
	2 or more	25 (56.8)
FF (degrees)	*N*	44
	Mean (SD)	125.23 (40.93)
	Median (IQR)	130 (90–160)
	Range	30–180
ABD (degrees)	*N*	44
	Mean (SD)	109.32 (43.03)
	Median (IQR)	95 (80–142.5)
	Range	20–180
ER (degrees)	*N*	44
	Mean (SD)	30 (17.98)
	Median (IQR)	30 (20–50)
	Range	− 10–60
Serum glucose concentration (mg/dL)	*N*	43
	Mean (SD)	100.65 (14.53)
	Median (IQR)	97 (91–107)
	Range	63–145

*BMI* body mass index, *NSAID* non-steroidal anti-inflammatory drug, *FF* flexion, *ABD* abduction, *ER* external rotation

**Fig. 1:**
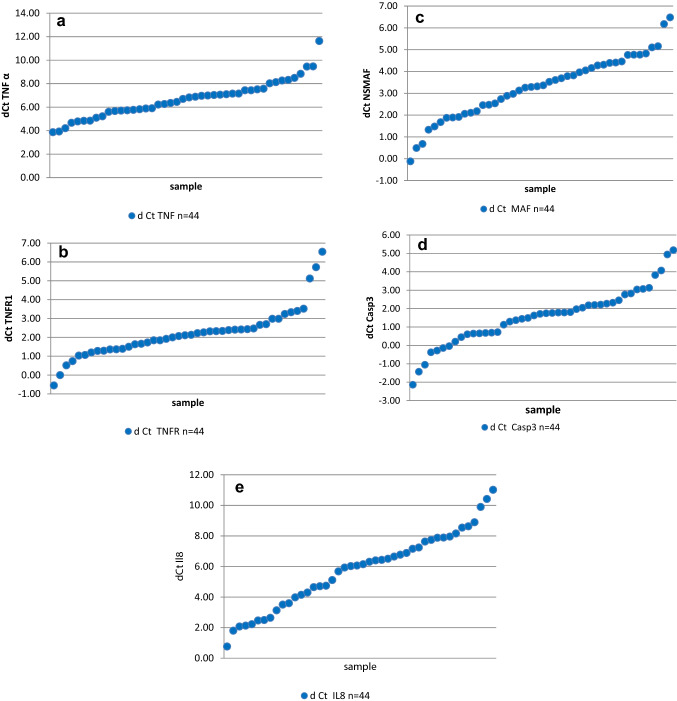
Relative TNFα mRNA (panel a), TNFR1 mRNA (panel b), NSMAF mRNA (panel c), Casp3 mRNA (panel d), IL-8 mRNA (panel e) expression for each participant in the study, ranked in ascending order of dCt values. The difference between the lowest and highest expression levels were between about 130-fold (TNFR1 mRNA) to more than 1000-fold (IL-8 mRNA)

The mean relative mRNA expression levels of the investigated genes (expressed as dCt) are shown in Table 2. These results were ranked in ascending order (Fig. 1a–e). We observed large variations in expression between study participants. The variations in expression were more than 250-fold for *TNFα*, about 130-fold for *TNFR1*, *NSMAF*, and *Casp3*, and more than 1000-fold for *IL-8*.

**Table 2:** Relative mRNA expression of genes related to TNF-α pathway in patients with rotator cuff tendinopathy

Gene	Unit/category	Relative expression (dCt)
*TNF*	*N*	44
	Mean (SD)	6.67 (1.59)
	Median (IQR)	6.77 (5.7–7.46)
	Range	3.87–11.63
*TNFR1*	*N*	44
	Mean (SD)	2.2 (1.31)
	Median (IQR)	2.12 (1.38–2.52)
	Range	− 0.55–6.54
*NSMAF*	*N*	44
	Mean (SD)	3.28 (1.46)
	Median (IQR)	3.34 (2.16–4.33)
	Range	− 0.12–6.48
*Casp3*	*N*	44
	Mean (SD)	1.56 (1.53)
	Median (IQR)	1.73 (0.65–2.28)
	Range	− 2.14–5.18
*IL8*	*N*	44
	Mean (SD)	5.76 (2.5)
	Median (IQR)	6.11 (3.88–7.66)
	Range	0.77–11.02

*TNF* tumour necrosis factor, *TNFR1* tumour necrosis factor receptor 1, *NSMAF* neutral sphingomyelinase activation associated factor, *Casp3* caspase 3, *IL-8* interleukin 8

We analysed whether any of the investigated genes showed correlated expression levels. The results (Pearson’s correlations) indicated correlated expression for all these genes (for all correlations *p* < 0.01).

The Mann–Whitney *U* test revealed that previously administered steroid therapy, medical history of the shoulder injury, and BMI did not influence the mRNA expression of: *TNFα* (*p* = 0.26, *p* = 0.16, *p* = 0.13, respectively), *TNFR1* (*p* = 0.35, *p* = 0.9, *p* = 0.81, respectively), *NSMAF* (*p* = 0.78, *p* = 0.88, *p* = 0.5, respectively), *Casp3* (*p* = 0.74, *p* = 0.18, *p* = 0.71, respectively), or *IL-8* (*p* = 0.48, *p* = 0.07, *p* = 0.27, respectively).

We found that the previous administration of NSAIDs, night pain, the presence of upper subluxation of the humeral head, and the number of damaged tendons were not significantly correlated with the mRNA expression of: *TNFα* (*p* = 0.99, *r* = 0.00; *p* = 0.57, *r* = 0.09; *p* = 0.09, *r* = − 0.26; *p* = 0.19, *r* = 0.2, respectively), *TNFR1* (*p* = 0.91, *r* = − 0.02; *p* = 0.24, *r* = 0.18; *p* = 0.2, *r* = 0.2; *p* = 0.66, *r* = 0.07, respectively), *NSMAF* (*p* = 0.87, *r* = 0.03; *p* = 0.62, *r* = 0.08; *p* = 0.8, *r* = − 0.04; *p* = 0.95, *r* = 0.01, respectively), *Casp3* (*p* = 0.8, *r* = 0.05; *p* = 0.82, *r* = 0.04; *p* = 0.77, *r* = 0.05; *p* = 75, *r* = − 0.05, respectively), or *IL-8* (*p* = 0.08, *r* = − 0.27; *p* = 0.49, *r* = 0.1; *p* = 0.62, *r* = − 0.08; *p* = 0.5, *r* = − 0.1, respectively).

We found significant, but weak correlations between the flexion (FF) and external rotation (ER) range of motion and mRNA expression of *IL-8* (*p* = 0.045, *r* = 0.304 and *p* = 0.043, *r* = 0.306, respectively), and between the abduction (ABD) range of motion and mRNA expression of *Casp3* (*p* = 0.024, *r* = 0.341).

## Discussion

RC tendinopathy is a common condition, and it is associated with impaired function. The aetiology and pathophysiology of this condition remain unclear, but new research and development of new diagnostic techniques have resulted in accumulating evidence in support of the role of inflammation in tendon injures. Inflammation is involved in the development, progression, and healing stages of tendon injuries (Abraham et al. 2017; Buhrmann et al. 2011; Dean et al. 2016). Millar et al. (2009) demonstrated that pro-inflammatory cytokines and apoptotic genes were significantly upregulated in a rodent model of tendinopathy, and they confirmed these results in humans. Other researchers proposed that pro-inflammatory cytokines, such as IL-1β could initiate tendinopathies by stimulating inflammation, apoptosis, and extracellular matrix degradation (Mobasheri and Shakibaei 2013). In the present study, we examined gene expression for selected pro-inflammatory factors, namely TNF-α, TNFR1, NSMAF, IL-8, and Casp3. TNF-α binds with a high affinity to TNFR1 and with a low affinity to TNFR2. TNFR1 is expressed in almost all cells types, and TNFR2 is expressed in only selected cells (Speeckaert et al. 2012). TNFR1 and TNFR2 activate different downstream transduction pathways (Speeckaert et al. 2012). The cytoplasmatic tail of TNFR1 consists of several functionally distinct domains. One leads to the activation of pro-inflammatory and apoptotic pathways through the formation of two molecular complexes. The first complex (complex 1) is involved in activating transcription factors, such as NF-κB, which in turn leads to the activation of genes encoding pro-inflammatory cytokines, including IL-8. The second complex (complex 2, also known as death-inducing signalling complex) leads to activation of the caspase cascade in mitochondrial-dependent and -independent manners (Chu 2013; Croft et al. 2012; Montfort et al. 2010; Neumeyer et al. 2006; Nikoletopoulou et al. 2013). Another domain of TNFR1 that might be involved in signal transduction is the neutral sphingomyelinase domain. This domain interacts with NSMAF (alias FAN), which is involved in both pro-inflammatory gene expression and caspase cascade activation (Montfort et al. 2010). We showed gene expression levels for several pro-inflammatory factors, and their mutual correlations supported the notion that inflammation played a role in the course of RC tendinopathy. However, we detected large variations in the degree of expression among individual patients. Some genes varied in expression by more than 1000-fold among our group of patients. These differences might result from different causative factors for RC tendinopathy; however, our correlation analysis showed that the medical history of the injury did not influence the expression levels of the investigated genes. Alternatively, the different expression levels might result from individuals in different stages of the inflammatory process, which is dynamic, or differences in individual immunological and inflammatory responses.

Regardless of the cause, these variations highlighted a knowledge gap about the sequence of events and the mediators of the inflammation accompanying tendinopathy (Dean et al. 2016). Clarification of the mechanism underlying RC tendinopathy could facilitate the development of therapeutic interventions. Currently, there are several therapeutical approaches in the strategy for treating RC tendinopathy. Surgery is performed for tears, tendon-to-bone ruptures, and when conservative treatment has failed; however, the success rate is moderate (Abraham et al. 2017; Chianca et al. 2018). Conservative therapy typically includes rest, physical therapy, and NSAIDs or steroids. Despite the fact that NSAIDs and steroids can relieve patient symptoms and pain, over long periods, they have provided limited benefits, mostly due to their deleterious effects on tissue healing (Abraham et al. 2017; Darrieutort-Laffite et al. 2019). Other therapies currently applied in RC tendinopathy include platelet-rich plasma injections, application of mesenchymal stem cells, autologous conditioned serum injections, or signalling inhibition. We need to mention, especially in terms of genes selected in our study, anti-TNF therapy. This therapy is recommended in Immune-mediated inflammatory diseases), namely spondyloarthritis, psoriasis, Crohn’s disease, and rheumatoid arthritis and has proven effective to reduce inflammation. However, the way TNF inhibitors (TNFi) affect the immune system in patients is not yet clear, especially due to the pleiotropic mode of TNF-α activity. Furthermore, the clinical efficacy of TNFi therapy is limited by a high rate of non-responsiveness (30–40%) and additionally it has been associated with infectious complications (Menegatti et al. 2019).

Mentioned above therapies recommended in RC tendinopathy are promising, and they might provide benefits, but the data regarding effectiveness, particularly in the long term, are controversial (Damjanov et al. 2018; Gulotta et al. 2011; Kim et al. 2019; Lin et al. 2020; Rynecki and Pereira 2018). Our data, which showed wide variations in the expression of pro-inflammatory factors, might partially explain these controversies. Immunomodulatory therapy should be adjusted to the current state of RC inflammation. However, our data indicated that this state could not be determined from the medical history or standard clinical examination results. Therefore, when possible, a molecular diagnostic test should be considered for determining the most appropriate, effective therapy for RC tendinopathy.

This study had some limitations. First, the patients enrolled in the study comprised only those referred to surgery. Second, we examined only gene expression, not the concentration of proteins in tendon tissues. Therefore, we did not consider the possibility of posttranslational changes. Third, we examined only the selected factors, namely those included in the TNF-α signalling pathway; however, other inflammatory mediators could be involved. Also the usage of steroids or NSAIDs could interfere with inflammatory gene expressions. In our study, we did not consider such clinical scales as ASES, DASH, SST, SPADI or others.

In conclusion, we confirmed that the expression levels of selected genes that encoded pro-inflammatory factors in the TNF-α signalling pathway were involved in the course of RC tendinopathy. However, the expression levels varied dramatically from patient to patient. Moreover, gene expression could not be predicted on the basis of a standard clinical examination. Our results suggested that the current inflammatory state should be considered for selecting the best therapy, and therefore, when possible, clinicians should consider a molecular diagnosis of inflammation in RC tendinopathy, and subsequent therapy decisions should be based on the results.

## Data Availability

Data are available upon request from the first author.
